# Associations between viewable greenspace and adolescent wellbeing in Wales.

**DOI:** 10.23889/ijpds.v7i3.1979

**Published:** 2022-08-25

**Authors:** Amy Mizen, James Rafferty, Emily Lowthian, Rowena Bailey, Ann John, Richard Fry, Lucy Griffiths

**Affiliations:** 1 Swansea University

## Objectives

Mental wellbeing can deteriorate throughout adolescence; females and children from low-income families more likely to experience mental health conditions. Views of greenspace from home positively impact cognition, but links with wellbeing has not been explored in children. We linked environment and survey data for 14 year olds in Wales, UK.

## Approach

Our cross-sectional study examined the relationship between views of greenspace and wellbeing for >1000 children aged 14 years living in Wales between 2015-2016. We linked data on views of greenspace from the home location with individual-level wellbeing and socio-demographic data in the SAIL Databank; a secure research environment. Our health outcome was derived from self-reported wellbeing measures in the Millennium Cohort Study. Views of greenspace were derived from LiDAR data and quantified on a continuous scale (0-1). We used Generalised Additive Models to investigate associations between views of greenspace and wellbeing; adjusting for factors such as parent wellbeing and deprivation.

## Results

Homes in coastal areas had larger views of greenspace than non-coastal residences. Individuals living in the most deprived areas had smaller views of greenspace (mean = 0.03) than least deprived (mean = 0.12). Overall, individuals living in detached homes had the greatest views of greenspace (0.4) and flats had the poorest views of greenspace (mean = 0.02). We will report our final regression analyses at the conference investigating the association between views of greenspace and adolescent wellbeing. Our models will be fully adjusted and sub-analyses will be stratified by gender and urban/rural status. We will also report findings on whether deprivation mediates for any relationships.

## Conclusion

Our study is the first to link objectively measured views of greenspace with wellbeing data for a national cohort. Our results can be used to develop interventions to support good wellbeing in adolescents. Further longitudinal research is required to investigate the causal pathways between views of greenspace and adolescent wellbeing.

